# Joint use of over- and under-sampling techniques and cross-validation for the development and assessment of prediction models

**DOI:** 10.1186/s12859-015-0784-9

**Published:** 2015-11-04

**Authors:** Rok Blagus, Lara Lusa

**Affiliations:** Institute for Biostatistics and Medical Informatics, University of Ljubljana, Vrazov trg 2, Ljubljana, Slovenia

**Keywords:** Prediction models, Class-imbalance, Random undersampling, Simple oversampling, SMOTE, Cross-validation, Overoptimism

## Abstract

**Background:**

Prediction models are used in clinical research to develop rules that can be used to accurately predict the outcome of the patients based on some of their characteristics. They represent a valuable tool in the decision making process of clinicians and health policy makers, as they enable them to estimate the probability that patients have or will develop a disease, will respond to a treatment, or that their disease will recur. The interest devoted to prediction models in the biomedical community has been growing in the last few years. Often the data used to develop the prediction models are class-imbalanced as only few patients experience the event (and therefore belong to minority class).

**Results:**

Prediction models developed using class-imbalanced data tend to achieve sub-optimal predictive accuracy in the minority class. This problem can be diminished by using sampling techniques aimed at balancing the class distribution. These techniques include under- and oversampling, where a fraction of the majority class samples are retained in the analysis or new samples from the minority class are generated. The correct assessment of how the prediction model is likely to perform on independent data is of crucial importance; in the absence of an independent data set, cross-validation is normally used. While the importance of correct cross-validation is well documented in the biomedical literature, the challenges posed by the joint use of sampling techniques and cross-validation have not been addressed.

**Conclusions:**

We show that care must be taken to ensure that cross-validation is performed correctly on sampled data, and that the risk of overestimating the predictive accuracy is greater when oversampling techniques are used. Examples based on the re-analysis of real datasets and simulation studies are provided. We identify some results from the biomedical literature where the incorrect cross-validation was performed, where we expect that the performance of oversampling techniques was heavily overestimated.

**Electronic supplementary material:**

The online version of this article (doi:10.1186/s12859-015-0784-9) contains supplementary material, which is available to authorized users.

## Background

In clinical research the goal is often to estimate the probability that patients have or will develop a disease, will respond to a treatment, or that their disease will recur; prediction models can be used to accurately predict the outcome of the patients based on some of their characteristics. Prediction models represent a valuable tool in the decision making process of clinicians and health policy makers and are extensively used in medicine [1], however the majority of prediction studies in high impact journals do not follow the current methodological recommendations, limiting their reliability and applicability [2].

Prediction models are often developed on class-imbalanced data: for example, data gathered from screening programs usually include few patients with the disease (minority class samples) and many healthy subjects (majority class samples). Such models tend to achieve poor predictive accuracy in the minority class [3]. Sampling methods are the most widely used strategy to improve the predictive accuracy of the minority class, their aim is to obtain a balanced distribution prior to building the prediction model. Undersampling techniques remove some of the majority class subjects, while oversampling methods generate additional minority class subjects based on the observed data. These techniques are also often applied in the field of bioinformatics [4–7].

Synthetic Minority Oversampling TEchnique (SMOTE [8]) is a sampling method that is widely used to improve the performance of the prediction models [9, 10]. SMOTE generates new minority class samples on a random point of the line joining a minority class sample and one of its nearest neighbors. Additionally, frequently a predefined proportion of majority class samples is randomly selected and discarded from the training set. In systematic studies it was observed that random undersampling tends to outperform SMOTE in most situations [11, 12]. However, others showed that on specific datasets SMOTE performed better than undersampling [13–15]. SMOTE was also evaluated for high-dimensional data, where the number of variables greatly exceeds the number of samples [16]. High-dimensional data are increasingly often used for developing the prediction models in medicine [17–19]. It was shown that SMOTE performs poorly in the high-dimensional setting when compared with random undersampling [16].

In the absence of an independent validation dataset, the performance of the prediction models on new samples is usually estimated using cross-validation (CV). In *k*-fold CV the dataset is divided into *k* parts, one part is withdrawn and used as a test set, the other *k*−1 parts are used to build the prediction model. The process is iterative: each of the *k* folds is used once as a test set and the performance of the classifier (prediction model) is obtained averaging the results. In order to correctly perform CV it is essential to observe the principle that all the steps involved in the building of the prediction model must be performed using only the training data. For this reason the sampling step should not be performed on the entire dataset, but instead only on the training set of each partition generated during the CV procedure. Failing to do so will produce unreliable and overoptimistic cross-validated estimates of the performance of the prediction model.

Although the importance of correct CV is well recognized in the statistical community [20], numerous papers where oversampling was not correctly implemented in CV can be found. For example, Naseriparsa and Kashani [13] investigated the usefulness of combining SMOTE with principal component analysis, Lopez-de-Uralde et al. [14] used SMOTE for the automatic morphological categorization of carbon black nano-aggregates and Taft et al. [5] applied SMOTE to improve adverse drug event predictive models in labor and delivery. All these papers showed that SMOTE improved the cross-validated accuracy of the prediction models; however, these cross-validated estimates are expected to be overoptimistic, as CV was used after SMOTE-augmenting the entire dataset and the SMOTE-sampling step was not included in CV. Similar incorrect uses of CV on oversampled data can be found in numerous papers (see [6, 15, 21], to name only some of the most recent examples).

Two groups have studied the over-optimism in the estimation of the prediction error due to incorrect CV [22, 23]. These works focused on the bias due to the omission of the variable selection step in CV and had a large impact on the quality of subsequent published research, especially when considering high-dimensional data.

Others showed that similar problems are encountered when classifier parameter tuning is based on minimizing cross-validated error rates, which is performed outside CV loop [24]. To our knowledge our study is the first to evaluate the bias due to incorrect CV for prediction models that use sampling techniques.

In this paper we illustrate the problems using publicly available datasets from the UCI machine learning repository [25] and gene expression microarray datasets. The results are explained also from a theoretic perspective as well as using a small simulation study. The implication of our results for practical predictive modeling with class-imbalanced data are discussed.

## Methods

We considered only prediction models for two classes, with *n*_*min*_ samples in the minority class and *n*_*maj*_ in the majority class, using classification trees (CART [26]). In CART the Gini index was used as a measure of node impurity, there had to be at least two samples in the node to attempt the partition of the data and the maximum depth of each tree was set to 30. The classifiers were fitted using the function rpart included in the rpart R package.

We used three types of sampling techniques to reduce the class-imbalance problem: random undersampling, simple oversampling and SMOTE. Sampling was performed before CV (incorrect analysis, Sampling followed by CV) or included in the CV procedure (correct analysis, CV includes Sampling). Six types of cross-validated performance measures were evaluated for each classifier. The results were evaluated using simulated and real class-imbalanced data.

All analyses were performed with R language for statistical computing (R version 3.0.3) [27].

### Evaluation of the cross-validated performance of the prediction model

We evaluated six cross-validated performance measures. Overall predictive accuracy (PA, defined as the proportion of correctly classified samples), predictive accuracy for the minority and for the majority class (PA_*min*_ and PA_*max*_, defined as PA evaluated using only minority or majority class samples, respectively), g-means $(\text {GM}=\sqrt {\text {PA}_{\textit {min}}\text {PA}_{\textit {max}}})$, area under the receiver operating characteristic (ROC) curve (AUC) ([28], chapter 4) and F_1_ measure $\mathrm {F}_{1}=\frac {2\cdot \text {Precision}\cdot \text {PA}_{\textit {min}} }{\text {Precision}+\text {PA}_{\textit {min}}} $ (where Precision is the proportion of samples that were correctly classified in the minority class, also known as minority class predictive value).

Cross-validated estimates of these performance measures provide nearly unbiased estimates of the values that would be obtained on independent samples.

### Simulated data

The aim of the simulations was to show how the use of correct and incorrect CV impacts the cross-validated performance measures. We used the setting where there is no real difference between the classes, i.e., when the developed prediction models are uninformative about the class membership of new samples.

In this case the correct value of AUC and GM is 0.50 and PA_*min*_+PA_*max*_=1; deviations from these values of the cross-validated measures indicate that the CV was not performed correctly. The term overoptimism will be used to indicate a positive bias in the estimation of the performance measures.

All variables were simulated independently from a Gaussian distribution with zero mean and unit variance. We varied the size of the dataset (*n*=100,500,1,000,10,000) and the number of variables (*p*=10,100), while the level of class-imbalance was kept fixed at $10\,\% (\frac {n_{\textit {min}}}{n}=0.1)$. Different number of CV folds was also considered (*k*=2, 5, 10). The results were averaged over 1,000 simulation runs.

### Real data

Ten publicly available datasets from the UCI machine learning repository [25] were used. The description of the datasets is given in Table 1. These datasets were selected as they exhibit various levels of class-imbalance, ranging from small (sonar dataset, 46.6 % minority samples) to large (ozone dataset, 2.9 % minority samples), they have very different sample size (ranging from 32 to 17,307 samples) and number of variables (from 5 to 72), and the difficulty of the classification task varies. All multi-class classification tasks were transformed into binary classification task by merging the classes; the name of the resulting minority class is reported in Table 1.

**Table 1:** Description of the datasets. Size of the dataset (*n*), number of variables (*p*), number of minority class samples (*n*_*min*_) and number of majority class samples (*n*_*maj*_)

Name	*n*	*p*	*n*_*min*_	*n*_*maj*_	*n*_*min*_ (%)	Name minority
Indian	768	8	268	500	34.9	*Positive*
Parkinson	195	22	48	147	24.6	*Healthy*
Hepatitis	155	19	32	123	20.6	*Dead*
Abalone	4,177	8	1,307	2,870	31.3	*Female*
Letter	17,307	16	689	16,618	3.4	*A*
Lung	32	56	9	23	28.1	*1*
Tae	151	5	49	102	32.4	*Low*
Breast	106	9	22	84	20.8	*Adi*
Sonar	208	60	97	111	46.6	*Rock*
Ozone	2,536	72	73	2,463	2.9	*Ozone day*
Sotiriou:er	99	7,650	34	65	34.3	*ER-*
Sotiriou:grade	99	7,650	45	54	45.5	*Grade 3*
Ivshina:er	245	22,283	34	211	13.9	*ER-*
Ivshina:grade	245	22,283	55	234	22.4	*Grade 3*
Wang:er	286	22,283	77	209	26.9	*ER-*
Wang:relapse	286	22,283	107	179	37.4	*Relapse*

Additionally, six high-dimensional classification tasks were considered in our analysis. We reanalyzed the breast cancer microarray gene expression data of Sotirou et al. [29], Wang et al. [30] and Ivshina et al. [31] considering the prediction of Estrogen receptor status (ER; all datasets), grade of the tumor (Grade; Ivshina and Sotiriou datasets) and relapse of the tumor (Wang dataset), see also Table 1. The data were preprocessed as described in the original publications. Missing data were present in the cDNA two-channel dataset [29]: the genes with more than 10 % of missing values were removed from the analysis and the remaining missing values were replaced with zeros. The 1,000 variables exhibiting the largest variance were pre-filtered and used for further analysis.

We performed 500 runs of 5−fold CV and reported the averaged results.

### Sampling techniques

In random undersampling *n*_*min*_ samples from the majority class were selected without replacement and combined with all minority class samples; the classifier was trained using the reduced and balanced dataset of size 2·*n*_*min*_.

In simple oversampling *n*_*maj*_ samples from the minority class were randomly selected with replacement and combined with the majority class samples to form the augmented and balanced dataset of size 2·*n*_*maj*_.

In SMOTE we generated 1, 2 or 5 new samples for each minority class sample; in the following these analyses are indicated as 100-, 200- and 500-SMOTE, respectively. The number of majority class samples retained in the analyses was equal to the number of newly generated minority class samples (undersampling fraction of 100 %); 5 nearest neighbors were used. For SMOTE we used the function SMOTE in the DMwR package [32] in R (with parameters *k*=5,*p* *e* *r* *c*.*u* *n* *d* *e* *r*=100,*p* *e* *r* *c*.*o* *v* *e* *r*=100,200,500). Under- and oversampling were programmed in R.

### Cross-validation

In *k*-fold CV the dataset was divided into *k* parts (folds), *k*−1 parts were used to build the prediction model, the remaining part was used to evaluate its performance. We used balanced folds, i.e., the number of samples included in each fold and the level of class-imbalance in each fold was approximately the same. The process was repeated *k* times so that each of the *k* folds was used once as a test set. The performance of the prediction model was obtained by averaging the results from the *k* folds.

**Fig. 1:**
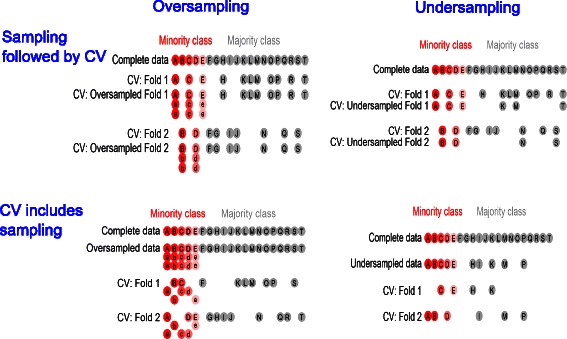
Combination of sampling and CV methods used in the simulations and real data analyses. CV includes Sampling (first row) constitutes the correct approach, while Sampling followed by CV (second row) is the incorrect approach. The samples included in the original dataset are indicated using upper cases, while their copies are indicated with lower cases

To evaluate the impact of resampling methods on CV results, two types of analyses were performed (graphically presented in Fig. 1 for 2-fold CV). In the correct CV the dataset was first split into *k* folds, the sampling method (over-, undersampling or SMOTE) was applied to the training set constituted of the *k*−1 folds and a reduced or augmented training set was obtained (procedure is indicated as CV includes Sampling, first row, in Fig. 1). In the incorrect CV different sampling techniques were first applied to the entire dataset and CV was applied to the over- or undersampled data, as described above (indicated as Sampling followed by CV, second row, in Fig. 1).

## Results

### Illustration of the problem

The problem when performing CV after simple oversampling (incorrect CV) is that the same samples can be included when building the prediction model and when evaluating its performance (Fig. 1, third panel). The probability that the same sample (either the original minority sample or its exact replica) is included in the training and test set can be obtained theoretically and is a function of the following: (1) sample size (*n*=*n*_*min*_+*n*_*maj*_; when the sample size is smaller the probability is larger), (2) proportion of samples included in the test set (*p*_*test*_; when the proportion is smaller the probability is larger), and (3) proportion of minority class samples,

(1) $$\begin{array}{@{}rcl@{}} 1-\frac{{n-n_{maj}/n_{min} \choose np_{test}-n_{maj}/n_{min}}}{{n-1 \choose np_{test}-1 }}, \end{array} $$

when *n* *p*_*test*_≥*n*_*maj*_/*n*_*min*_ and one otherwise.

**Fig. 2:**
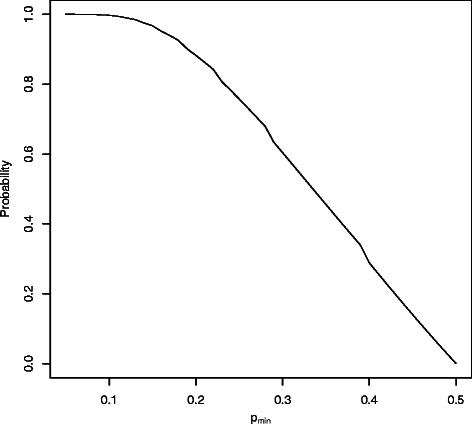
Probability that at least one of the replicas of a sample included in the test fold is included also in the training fold, as a function of the proportion of minority class samples (*p*_*min*_). The figure shows how the probability that a test sample has a replica in the learning fold depends on the level of class-imbalance (*p*_*min*_) in a dataset with *n*=100 samples when using 2 fold CV

As an illustration, we graphically show in Fig. 2 how the probability that a test (left-out) sample has a replica in the learning fold depends on the level of class-imbalance in a dataset with *n*=100 samples when 2-fold split is used (*p*_*test*_=0.5). The probability is very large for large levels of class-imbalance and approaches zero when the class distribution is more balanced.

In practice having large probabilities that replicas of test samples are included in the learning folds constitutes a problem. When the same sample is used to build the prediction rule and to evaluate its performance, the estimate of its performance is overoptimistic as it is obviously easier to correctly predict the class of the samples that were already used in the training phase due to over-fitting. This problem is illustrated from a more theoretic perspective in Additional file 1, where we consider one nearest neighbor classifier (1-NN, [33]) in combination with random undersampling or simple oversampling.

Next, we used simulated data to show how the incorrect CV (sampling followed by CV) can lead to invalid conclusions focussing on the case where the prediction models are uninformative and the correct values of the performance measures are known (AUC=GM=0.5,PA_*min*_+PA_*max*_=1). See the Methods section for more details.

**Fig. 3:**
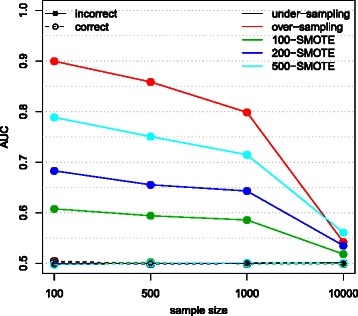
Cross-validated AUC for different sample sizes and classification rules obtained on simulated data. AUC obtained with different classification rules for simulated data with 10 variables (simulated independently from a Gaussian distribution with zero mean and unit variance) and 2 CV folds. There were *n*=100, 500, 1,000 and 100,00 samples

Here we show the results for the situation where the number of folds was set to 2 and there were 10 variables. The cross-validated AUC obtained for different values of *n* are shown in Fig. 3, exact numerical results for *n*=1,000 are shown in Table 2.

**Table 2:** Accuracy measures for simulated data. Accuracy measures obtained with different classification rules for simulated data with 10 variables (simulated independently from a Gaussian distribution with zero mean and unit variance), 1,000 samples and 2 CV folds

	PA	PA_min_	PA_maj_	GM	F_1_	AUC
Under (incorrect)	0.5003	0.5015	0.4991	0.4978	0.4996	0.5005
Under (correct)	0.4999	0.5009	0.4998	0.4985	0.1668	0.5003
Over (incorrect)	0.7624	0.8438	0.6809	0.7575	0.7801	0.7986
Over (correct)	0.7191	0.2254	0.7740	0.4151	0.1380	0.5004
100-SM (incorrect)	0.6422	0.7492	0.4281	0.5644	0.7359	0.5858
200-SM (incorrect)	0.6565	0.7462	0.5220	0.6230	0.7224	0.6431
500-SM (incorrect)	0.7012	0.7715	0.6168	0.6892	0.7378	0.7148
100-SM (correct)	0.4520	0.5582	0.4402	0.4940	0.1692	0.4993
200-SM (correct)	0.5294	0.4629	0.5368	0.4966	0.1643	0.4998
500-SM (correct)	0.5997	0.3757	0.6245	0.4824	0.1579	0.5007

The cross-validated AUC was equal to 0.5 for all prediction models when the correct CV was performed. The cross-validated AUC obtained with the incorrect CV was equal to 0.5 for undersampling, while it was substantially overestimated when data were oversampled or when SMOTE was used to generate synthetic samples. For SMOTE the overoptimism of AUC (positive bias) was larger when a larger fraction of synthetic samples were generated and for smaller datasets; the bias obtained with oversampling was even larger. For example, when the dataset consisted of 100 samples the difference between AUC obtained with the incorrect and the correct CV was 0.40, 0.29, 0.18 and 0.11 for oversampling, 500-SMOTE, 200-SMOTE and 100-SMOTE, respectively. Similar conclusions would be reached analyzing the other performance measures reported in Table 2.

Simulation results obtained in other settings are reported in the Additional file 2. In brief, we observed that increasing the number of variables and the number of CV folds slightly increased the overoptimism caused by the incorrect CV.

### Results on real data

**Fig. 4:**
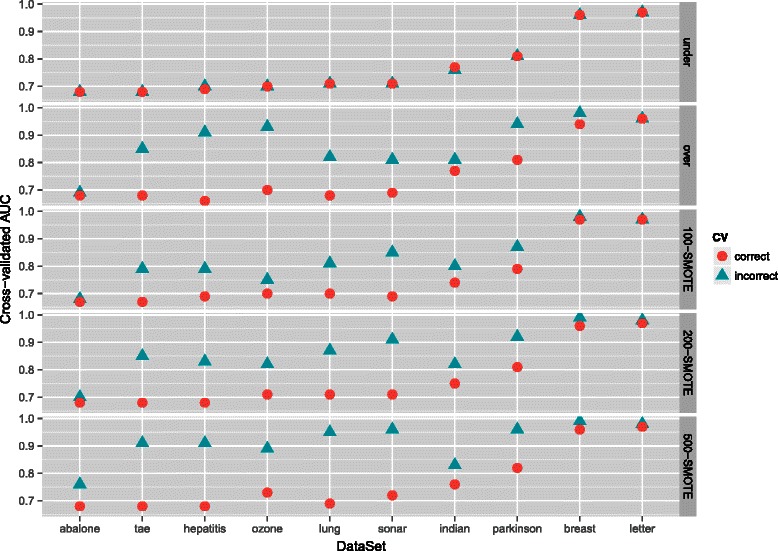
Cross-validated AUC for different UCI datasets. Datasets are ordered by their AUC obtained by correct CV

**Fig. 5:**
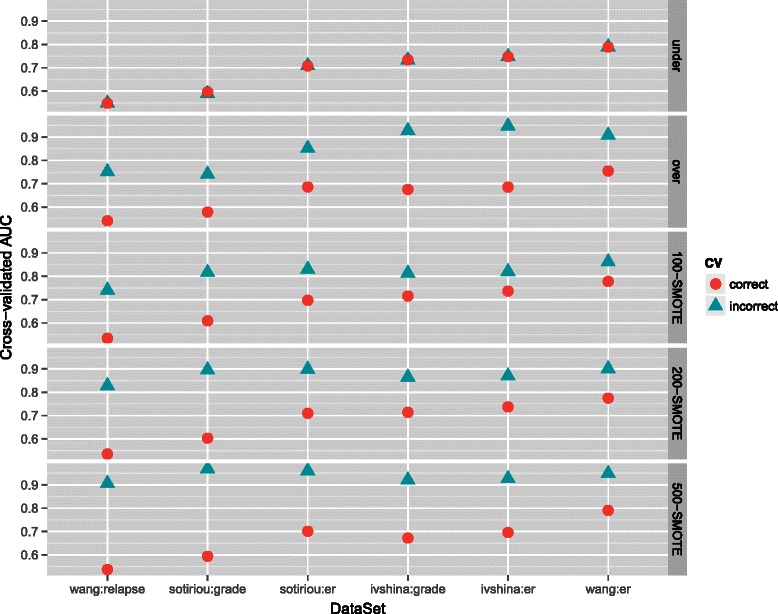
Cross-validated AUC for different gene expression microarray datasets datasets. Datasets are ordered by their AUC obtained by correct CV

Like in the simulated example the correct and the incorrect CV are compared on each dataset. See the Methods section for more details. Here we report the results for AUC graphically in Fig. 4 (UCI datasets) and Fig. 5 (gene expression microarray datasets); exact AUC, GM and F_1_-measure are reported in Additional file 3.

The results when performing the correct and the incorrect CV were the same when the datasets were undersampled, thus there was no bias when performing the incorrect CV (Figs. 4 and 5; Additional file 3). On the other hand, there was significant overoptimism in the cross-validated performance measures when performing the incorrect CV in combination with oversampling or SMOTE; the bias was larger when more synthetic samples were generated with SMOTE.

The bias was especially large for the smaller datasets, as for example the lung dataset where the difference between AUC obtained with the incorrect and the correct CV was as large as 0.14 with oversampling and 0.23 with 500-SMOTE. The difference between AUC obtained with the incorrect and the correct CV was however very small for very large datasets; the bias for the abalone dataset and the letter dataset for example was only marginal. This is in line with our simulated example where we observed that the overoptimism due to the incorrect CV is smaller when the dataset is larger. The bias was also only marginal when the prediction task was very easy, as in the breast dataset, where very accurate predictions could be obtained with random undersampling.

A similar behavior was observed also when considering gene expression data, but in this case the over-optimism when performing the incorrect CV in combination with oversampling or SMOTE was even larger. For example, AUC obtained with the correct CV with 500-SMOTE was 0.54 and with the incorrect CV the AUC was 0.91 for the wang:relapse dataset.

These results clearly show that the incorrect CV favors oversampling techniques as they appear to perform much better than random undersampling. However, when the correct CV is used, we obtained consistent results regardless of the sampling method applied.

## Discussion

In this paper we addressed the importance of correct CV for the assessment of the performance of the prediction models in medicine when some under- or oversampling method is used to improve the predictive accuracy for the minority class. There are some published studies in the field of biomedical informatics where under- or oversampling techniques are applied to the entire dataset and then CV is used on these modified datasets to estimate the performance of the prediction model. Our results using simulated data show that this type of incorrect CV leads to biased conclusions: oversampling techniques unjustifiably appear to perform better than undersampling techniques.

We explained that the reason for this bias is that oversampling techniques generate minority samples that are more similar or even identical to the original minority class samples and are hence easier to be correctly classified. This leads to overoptimistic cross-validated estimates of the accuracy of the minority class, while the accuracy for the majority class remains large due to the class-imbalance bias. Undersampling techniques, on the other hand, do not suffer from such bias and therefore unjustifiably appear to perform worse than oversampling techniques when evaluated with the incorrect CV. We observed that when the CV was performed correctly, i.e., the dataset was first split into *k* folds and then under- or oversampling techniques were applied only to *k*−1 folds used for training the classifier, the under- and oversampling techniques that we considered perform very similarly. These results are further verified and illustrated by using 10 publicly available datasets from the UCI repository and 6 gene expression microarray datasets, with varying degree of class-imbalance and where the differences between the classes were moderate or large.

The practical implication of these results for prediction models in medicine is twofold. The performance of the predictive model can be much worse when used on independent set of data than suggested by the incorrect CV. Specifically, the accuracy for the minority class subjects from the independent dataset will be much worse than suggested by the incorrect analysis. In practice this can have large negative consequences as it would mean that a larger proportion of subjects that have a disease will be incorrectly predicted to be healthy than suggested by the incorrect CV. Another implication is that the performance of the prediction model could be improved by using a different sampling technique than suggested by the incorrect analysis. Consider the UCI hepatitis data set as an example. The incorrect CV would suggest that oversampling is the most appropriate sampling technique for this dataset. However, the correct CV actually shows that this is the least appropriate technique for this dataset and that much better performance of the prediction model can be obtained by using undersampling. Even more extreme differences were observed for high-dimensional data.

It should be noted that the resistance to the incorrect CV observed for random undersampling does not apply to all undersampling techniques. To name an example from the field of bioinformatics, Rahman and Davis [21] proposed a cluster based undersampling technique to balance cardiovascular data. In their approach the majority class samples are clustered into 3 clusters by using K-means clustering and then these clusters are randomly undersampled and combined with all minority samples to obtain 3 datasets. Their results show a 22 percentage points increase in accuracy of this approach when compared with random undersampling. The problem, however, is that in their analysis the CV is applied after augmenting the dataset. We believe that this result is invalid as their method considers only majority class samples from the same cluster that are more similar to each other and it is hence easier to correctly classify them. Therefore, such analysis suffers from the same overoptimism as was described for the oversampling techniques. Special care is therefore needed also with undersampling techniques which generate datasets where minority class samples are, after reducing the dataset, more similar to each other. Such examples are NearMiss and the “most distant” undersampling techniques [34], where the use of incorrect CV could also lead to invalid conclusions.

## Conclusion

Researchers proposing new under- or oversampling techniques or researchers applying these techniques to improve the performance of prediction models that use CV to evaluate their models, should always include the sampling step in the CV loop, as their conclusions can otherwise be strongly misleading. Special care is also needed in the review process where the reviewers should always check if the correct CV was performed. It is also important that the researchers provide a clear and exact description of how the analysis was performed, as it is currently often impossible to say with certainty if the correct CV was performed or not. Attention is also needed when evaluating the effectiveness of the prediction models that were already proposed in the literature as there are numerous examples where the performance of these models was not estimated correctly.

## Additional files

Additional file 1
**Effect of the incorrect CV on 1-NN.** In the Additional file we illustrate the problem that was presented in the main text by considering one nearest neighbor classifier (1-NN) in combination with random undersampling or simple oversampling. (PDF 61.3 Kb)

Additional file 2
**Results using simulated data (3 figures).** In the Additional file we report the performance measures (AUC, GM and F_1_-measure) for different number of simulated variables (*p*), sample sizes (*n*) and CV folds (*k*). (PDF 151 Kb)

Additional file 3
**Results using real data (6 tables).** In the Additional file we present the performance measures (AUC, GM and F_1_-measure) obtained by reanalyzing UCI and gene expression microarray datasets. (PDF 109 Kb)

## Abbreviations

PA: predictive accuracy
SMOTE: synthetic minority oversampling technique
CV: cross-validation
AUC: area under the receiver operating characteristic curve
GM: geometric means of class-specific predictive accuracies
