# Supplementary material for: Joint use of over- and under-sampling techniques and cross-validation for the development and assessment of prediction models
Source: BMC Bioinformatics. 2015 Nov 4;16:363. doi: 10.1186/s12859-015-0784-9 (PMC4634915; doi:10.1186/s12859-015-0784-9)
Supplement: Additional file 1 — Effect of the incorrect CV on 1-NN. In the Additional file we illustrate the problem that was presented in the main text by considering one nearest neighbor classifier (1-NN) in combination with random undersampling or simple oversampling. (PDF 61.3 Kb) [file 12859_2015_784_MOESM1_ESM.pdf]

Effect of the incorrect cross-validation on 1-NN

Here we illustrate the problem that was presented in the main text by considering one nearest neighbor classifier (1-NN) in combination with random under-sampling or simple over-sampling. This classification rule is used as the expected bias due to the incorrect analysis can be easily understood intuitively. We will intuitively show that performing the incorrect CV (Sampling followed by CV) produces overoptimistic cross-validated performance measures when random under-sampling is used, while the estimates remain unbiased when using random under-sampling. We assume that the classification task is completely uninformative, i.e. none of the measured variables differs in the two classes. The probability that a new sample is classified in the minority class is in this setting simply the proportion of the minority class samples in the training set; this probability does not depend on the choice of the distance metric used to derive 1-NN. Suppose that our dataset contains 10 minority and 90 majority class samples.

The samples are divided into 2 folds so that each fold contains exactly 5 minority and 45 majority class samples; one fold is used to train the classifier and the other to evaluate its accuracy. If the training fold is under-sampled in order to obtain a class balanced distribution, we are left with 5 samples from each class. The probability that the minority class sample from the test fold is correctly classified is therefore $5/10 = 0.5$ and can be compared with the probability of $5/50 = 0.1$ if under-sampling had not been performed. When the dataset is first under-sampled in order to have 10 samples from each class, the probability that a minority class sample from the test fold is correctly classified in the minority class is still $5/10 = 0.5$, hence there is no bias when performing the incorrect cross-validation.

Assume again that the dataset is first divided in two parts so that there are 5 minority and 45 majority class samples in each fold. After using simple over-sampling the training fold contains 9 exact replicas of each minority class sample and 45 majority class samples. The probability that the minority class sample from the test fold is correctly classified is however only $5/50 = 0.1$ as in this setting all replicas count only as one sample (this holds as the replicas populate exactly the same spot in the feature space). Consider now the situation where the entire dataset is first over-sampled so that there are 90 minority (9 exact replicas of each minority class sample) and 90 majority class samples. Assume that after dividing the augmented dataset into a training and a test fold, there are 5 original minority class samples, 40 replicas (4 for each original minority class sample) and 45 majority class samples in each fold; the test fold therefore contains minority class samples that are exact replicas of some samples in the training fold. The probability that a minority sample from the test fold is correctly classified is equal to 1, as its exact replica from the training set is its nearest neighbor and is hence correctly classified. The probability that the majority sample from the test fold is correctly classified is in this setting $45/50 = 0.9$ as all replicas of the same minority class sample in the training fold count only as one sample. It is clear that the accuracy of 1-NN is in this case severely overestimated.
