# Supplementary material for: Joint use of over- and under-sampling techniques and cross-validation for the development and assessment of prediction models
Source: BMC Bioinformatics. 2015 Nov 4;16:363. doi: 10.1186/s12859-015-0784-9 (PMC4634915; doi:10.1186/s12859-015-0784-9)

AUC obtained on simulated data

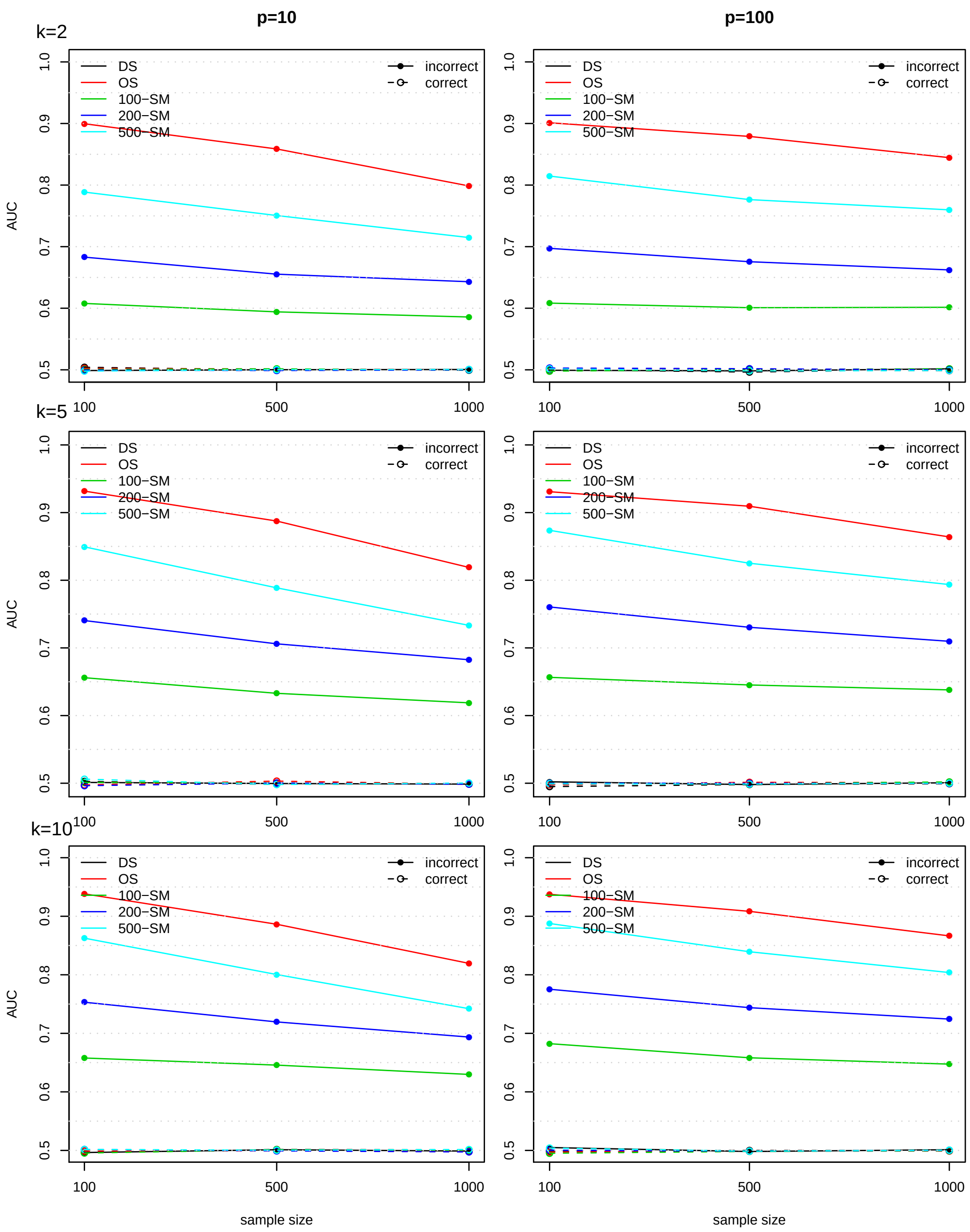

GM obtained on simulated data

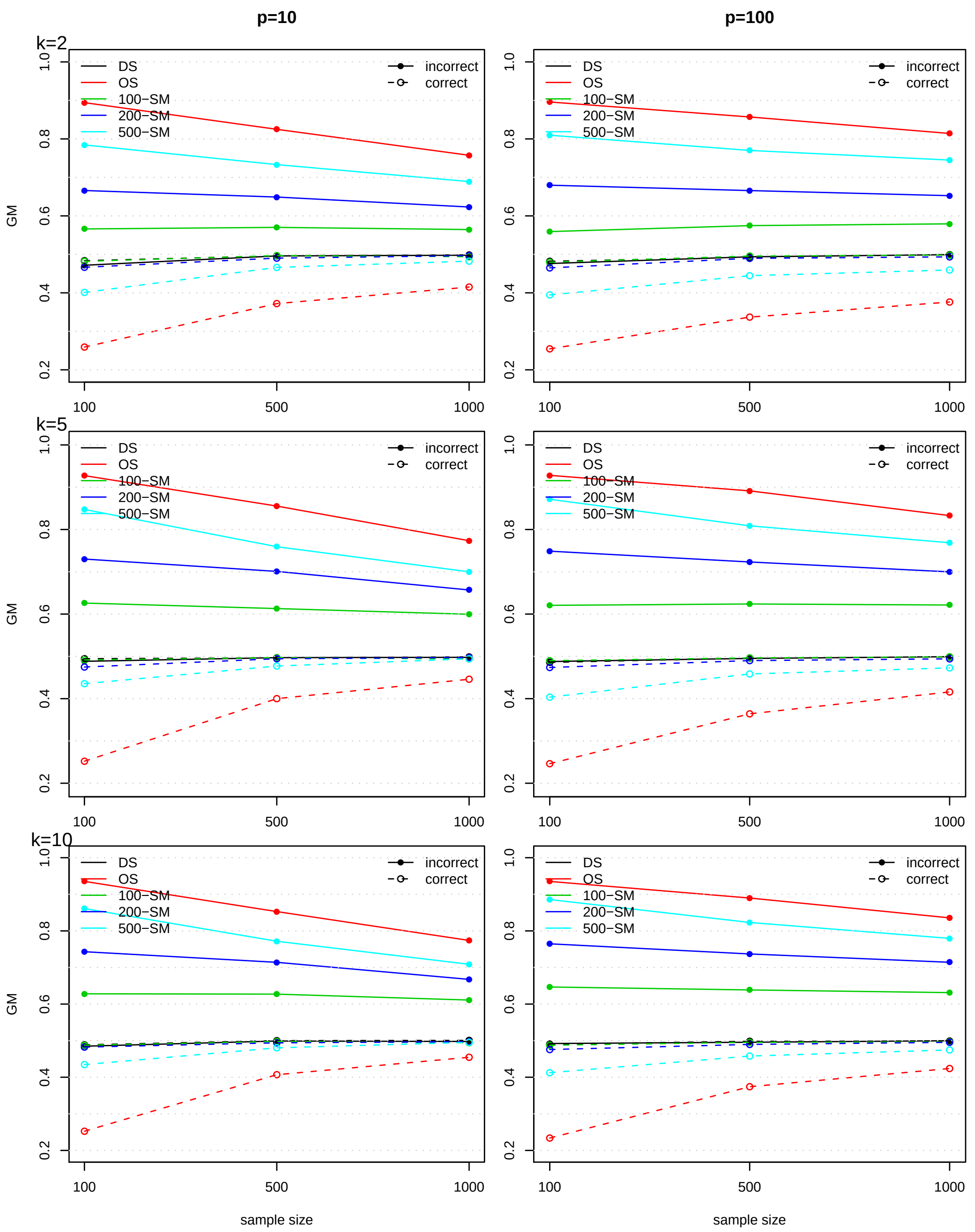

F_1-measure obtained on simulated data

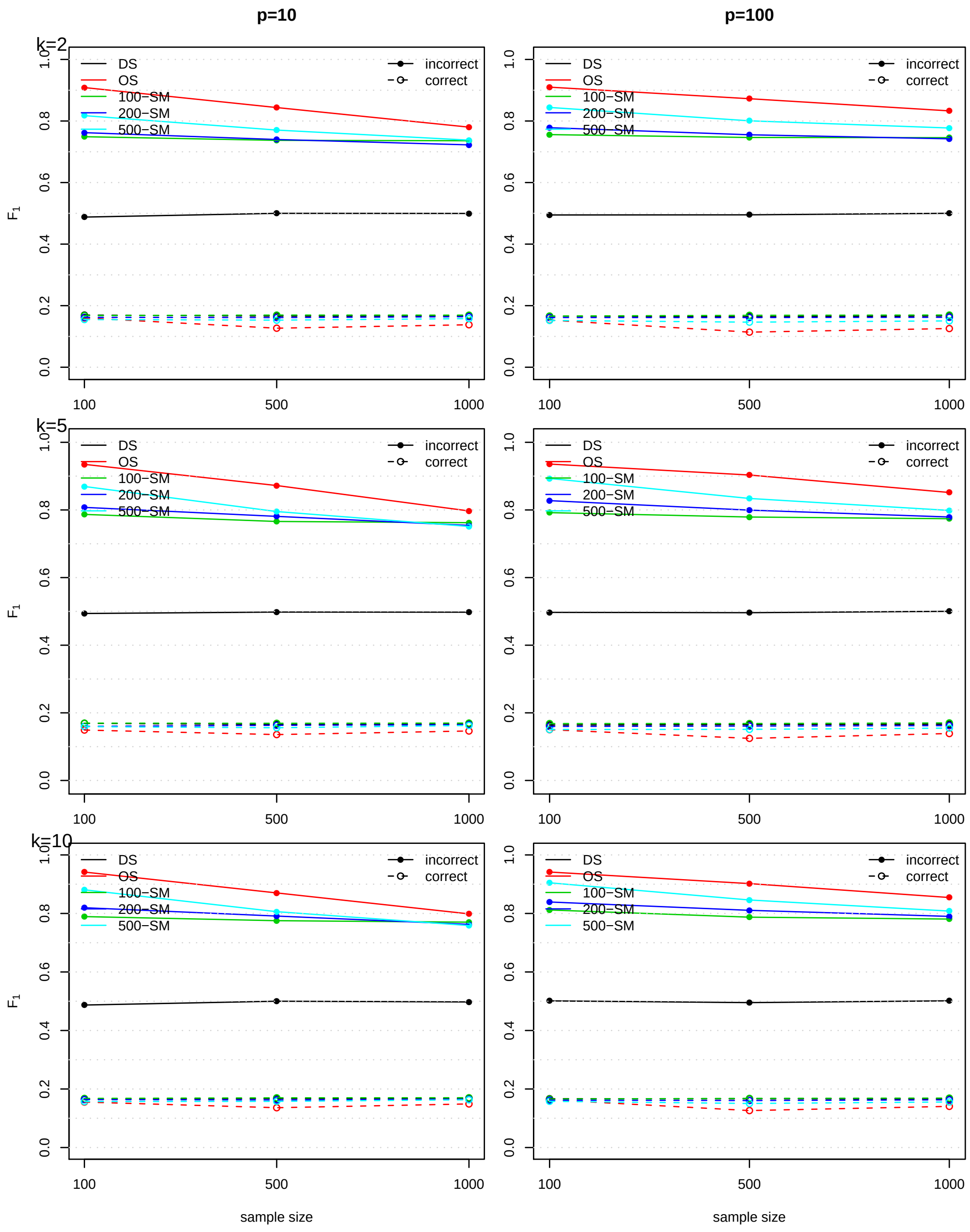

Supplement: Additional file 2 — Results using simulated data (3 figures). In the Additional file we report the performance measures (AUC, GM and F1-measure) for different number of simulated variables (p), sample sizes (n) and CV folds (k). (PDF 151 Kb) [file 12859_2015_784_MOESM2_ESM.pdf]
