# Supplementary material for: Joint use of over- and under-sampling techniques and cross-validation for the development and assessment of prediction models
Source: BMC Bioinformatics. 2015 Nov 4;16:363. doi: 10.1186/s12859-015-0784-9 (PMC4634915; doi:10.1186/s12859-015-0784-9)
Supplement: Additional file 3 — Results using real data (6 tables). In the Additional file we present the performance measures (AUC, GM and F1-measure) obtained by reanalyzing UCI and gene expression microarray datasets. (PDF 109 Kb) [file 12859_2015_784_MOESM3_ESM.pdf]

AUC obtained on real datasets

	indian	parkinson	hepatitis	abalone	letter	lung	tae	breast	sonar	ozone	**mean**
under (incorrect)	0.76	0.81	0.70	0.68	0.97	0.71	0.68	0.96	0.71	0.70	**0.77**
under (correct)	0.77	0.81	0.69	0.68	0.97	0.71	0.68	0.96	0.71	0.70	**0.77**
over (incorrect)	0.81	0.94	0.91	0.69	0.96	0.82	0.85	0.98	0.81	0.93	**0.87**
over (correct)	0.77	0.81	0.66	0.68	0.96	0.68	0.68	0.94	0.69	0.70	**0.76**
100-SMOTE (incorrect)	0.80	0.87	0.79	0.68	0.97	0.81	0.79	0.98	0.85	0.75	**0.83**
200-SMOTE (incorrect)	0.82	0.92	0.83	0.70	0.98	0.87	0.85	0.99	0.91	0.82	**0.87**
500-SMOTE (incorrect)	0.83	0.96	0.91	0.76	0.98	0.95	0.91	0.99	0.96	0.89	**0.91**
100-SMOTE (correct)	0.74	0.79	0.69	0.67	0.97	0.70	0.67	0.97	0.69	0.70	**0.76**
200-SMOTE (correct)	0.75	0.81	0.68	0.68	0.97	0.71	0.68	0.96	0.71	0.71	**0.77**
500-SMOTE (correct)	0.76	0.82	0.68	0.68	0.97	0.69	0.68	0.96	0.72	0.73	**0.77**

Table 1: AUC obtained on various UCI datasets. The table reports AUC averaged over 500 runs of 5 fold cross-validation for the correct and the incorrect cross-validation. See the Methods section for more details.

	sotiriou:er	sotiriou:grade	ivshina:er	ivshina:grade	wang:er	wang:relapse	**mean**
under (incorrect)	0.71	0.59	0.75	0.73	0.79	0.55	**0.69**
under (correct)	0.71	0.60	0.75	0.73	0.79	0.55	**0.69**
over (incorrect)	0.85	0.74	0.95	0.93	0.91	0.75	**0.85**
over (correct)	0.69	0.58	0.69	0.68	0.76	0.54	**0.65**
100-SMOTE (incorrect)	0.83	0.82	0.82	0.81	0.86	0.74	**0.81**
200-SMOTE (incorrect)	0.90	0.90	0.87	0.86	0.90	0.83	**0.88**
500-SMOTE (incorrect)	0.96	0.97	0.93	0.92	0.95	0.91	**0.94**
100-SMOTE (correct)	0.70	0.61	0.74	0.72	0.78	0.53	**0.68**
200-SMOTE (correct)	0.71	0.60	0.74	0.71	0.78	0.54	**0.68**
500-SMOTE (correct)	0.70	0.59	0.70	0.67	0.79	0.54	**0.67**

Table 2: AUC obtained on various gene expression microarray datasets. The table reports AUC averaged over 500 runs of 5 fold cross-validation for the correct and the incorrect cross-validation. See the Methods section for more details.

GM obtained on real datasets

	indian	parkinson	hepatitis	abalone	letter	lung	tae	breast	sonar	ozone	**mean**
under (incorrect)	0.73	0.81	0.69	0.64	0.95	0.71	0.67	0.96	0.71	0.73	**0.76**
under (correct)	0.73	0.81	0.69	0.65	0.95	0.71	0.67	0.96	0.71	0.73	**0.76**
over (incorrect)	0.77	0.92	0.90	0.65	0.94	0.80	0.81	0.98	0.81	0.92	**0.85**
over (correct)	0.73	0.81	0.65	0.65	0.94	0.68	0.65	0.94	0.70	0.74	**0.75**
100-SMOTE (incorrect)	0.75	0.86	0.78	0.59	0.95	0.80	0.78	0.98	0.85	0.77	**0.81**
200-SMOTE (incorrect)	0.78	0.92	0.84	0.63	0.96	0.86	0.83	0.99	0.90	0.82	**0.85**
500-SMOTE (incorrect)	0.80	0.95	0.90	0.70	0.96	0.95	0.87	0.99	0.94	0.87	**0.89**
100-SMOTE (correct)	0.69	0.79	0.69	0.59	0.94	0.70	0.66	0.96	0.69	0.72	**0.74**
200-SMOTE (correct)	0.72	0.81	0.69	0.62	0.95	0.70	0.66	0.96	0.72	0.72	**0.76**
500-SMOTE (correct)	0.73	0.82	0.66	0.64	0.95	0.69	0.64	0.96	0.72	0.71	**0.75**

Table 3: GM obtained on various UCI datasets. The table reports GM averaged over 500 runs of 5 fold cross-validation for the correct and the incorrect cross-validation. See the Methods section for more details.

	sotiriou:er	sotiriou:grade	ivshina:er	ivshina:grade	wang:er	wang:relapse	**mean**
under (incorrect)	0.71	0.59	0.75	0.73	0.79	0.55	**0.68**
under (correct)	0.71	0.59	0.75	0.73	0.79	0.55	**0.69**
over (incorrect)	0.85	0.74	0.94	0.92	0.91	0.74	**0.85**
over (correct)	0.67	0.56	0.65	0.66	0.78	0.52	**0.64**
100-SMOTE (incorrect)	0.83	0.81	0.82	0.81	0.86	0.74	**0.81**
200-SMOTE (incorrect)	0.90	0.90	0.87	0.86	0.90	0.82	**0.88**
500-SMOTE (incorrect)	0.96	0.96	0.93	0.92	0.94	0.89	**0.93**
100-SMOTE (correct)	0.69	0.60	0.74	0.71	0.78	0.53	**0.68**
200-SMOTE (correct)	0.71	0.60	0.73	0.71	0.79	0.54	**0.68**
500-SMOTE (correct)	0.71	0.60	0.71	0.69	0.80	0.54	**0.67**

Table 4: GM obtained on various gene expression microarray datasets. The table reports GM averaged over 500 runs of 5 fold cross-validation for the correct and the incorrect cross-validation. See the Methods section for more details.

F_1-measure obtained on real datasets

	indian	parkinson	hepatitis	abalone	letter	lung	tae	breast	sonar	ozone	**mean**
under (incorrect)	0.73	0.81	0.70	0.70	0.95	0.73	0.68	0.96	0.72	0.73	**0.77**
under (correct)	0.77	0.67	0.48	0.56	0.62	0.58	0.57	0.90	0.73	0.84	**0.67**
over (incorrect)	0.77	0.93	0.91	0.71	0.94	0.84	0.82	0.98	0.80	0.92	**0.86**
over (correct)	0.78	0.72	0.49	0.56	0.62	0.54	0.54	0.89	0.74	0.93	**0.68**
100-SMOTE (incorrect)	0.68	0.92	0.86	0.83	0.97	0.88	0.86	0.99	0.81	0.71	**0.85**
200-SMOTE (incorrect)	0.74	0.94	0.87	0.79	0.97	0.90	0.87	0.99	0.88	0.78	**0.87**
500-SMOTE (incorrect)	0.78	0.96	0.91	0.77	0.97	0.96	0.88	0.99	0.94	0.86	**0.90**
100-SMOTE (correct)	0.70	0.64	0.48	0.56	0.53	0.57	0.57	0.91	0.68	0.81	**0.65**
200-SMOTE (correct)	0.74	0.68	0.49	0.56	0.59	0.58	0.56	0.92	0.72	0.86	**0.67**
500-SMOTE (correct)	0.77	0.71	0.48	0.54	0.64	0.56	0.52	0.92	0.73	0.90	**0.68**

Table 5: F_1-measure obtained on various UCI datasets. The table reports F_1-measure averaged over 500 runs of 5 fold cross-validation for the correct and the incorrect cross-validation. See the Methods section for more details.

	sotiriou:er	sotiriou:grade	ivshina:er	ivshina:grade	wang:er	wang:relapse	me
under (incorrect)	0.71	0.59	0.75	0.73	0.79	0.55	0.69
under (correct)	0.76	0.62	0.45	0.51	0.67	0.47	0.58
over (incorrect)	0.84	0.72	0.95	0.93	0.91	0.76	0.85
over (correct)	0.80	0.65	0.46	0.50	0.69	0.41	0.58
100-SMOTE (incorrect)	0.77	0.75	0.89	0.88	0.91	0.83	0.84
200-SMOTE (incorrect)	0.88	0.87	0.90	0.90	0.92	0.86	0.89
500-SMOTE (incorrect)	0.95	0.96	0.93	0.92	0.95	0.89	0.93
100-SMOTE (correct)	0.73	0.59	0.43	0.48	0.65	0.49	0.56
200-SMOTE (correct)	0.77	0.61	0.46	0.50	0.68	0.47	0.58
500-SMOTE (correct)	0.78	0.61	0.47	0.49	0.70	0.46	0.58

Table 6: F_1-measure obtained on various gene expression microarray datasets. The table reports F_1-measure averaged over 500 runs of 5 fold cross-validation for the correct and the incorrect cross-validation. See the Methods section for more details.
